# Probing Behavior of Apterous and Alate Morphs of two Potato—Colonizing Aphids

**DOI:** 10.1673/031.011.16401

**Published:** 2011-12-02

**Authors:** Sébastien Boquel, Philippe Giordanengo, Arnaud Ameline

**Affiliations:** ^1^ Université de Picardie Jules Verne, Biologie des Plantes et Contrôle des Insectes Ravageurs, 33 rue Saint Leu, 80039 Amiens Cedex, France; ^2^ GIE, Station de Recherche et de Création Variétale du Comité Nord, 76110 Bretteville-du-Grand-Caux, France

**Keywords:** Aphididae, electrical penetration graph, *Macrosiphum euphorbiae*, *Myzus persicae*, *Solanum tuberosum*, virginoparae

## Abstract

Secondary host plant colonization by aphids involves alate and apterous morphs to spread in the population at a large scale by flying or, at a finer one, by walking. *Macrosiphum euphorbiae* Thomas (Hemiptera: Aphididae) and *Myzus persicae* Sulzer (Hemiptera: Aphididae) are two polyphagous aphids that cause serious losses on many crops, particularly on potato, *Solanum tuberosum* L. (Solanales: Solanaceae). When settlement of virginoparous alate aphids occurs, apterous individuals are produced and spread within the potato field. As these two potato colonizers originate from different areas and show different body length, this study compared probing behaviors of virginoparous alate and apterous *M. persicae* and *M. euphorbiae* on one of their secondary host plants, *Solanum tuberosum*. Non—choice bioassays and electrical penetration graph (EPG) recordings were performed. Most *M. euphorbiae* of the two morphs rapidly accepted potato plants and exhibited long duration of probing, phloem sap salivation, and ingestion phases. In contrast, at the end of the experiment, most alates of *M. persicae* left the potato leaflet after brief gustative probes. Moreover, EPG experiments showed that the main difference between both morphs of the two species concerned the xylem ingestion parameter. Differences between species were also reported, such as an increased total duration of probing in both morphs and enhanced phloem ingestion duration in apterous *M. euphorbiae*. All the differences highlighted in this study are discussed according to the variations observed in aphid body size and to their historical association with *Solanum* species.

## Introduction

Sexual and parthenogenetic reproduction, which occur during the heteroecic aphid life cycle, necessitate seasonal migration between at least two host plants. The primary host plant is colonized in autumn by gynoparae for sexual reproduction, while the secondary host is colonized during spring and summer by virginoparae that reproduce parthenogenetically. Apterous and alate females are respectively adapted to either reproduction or host—plant colonization strategies related to morphological or physiological traits (Pickett et al. 1992; Park and Hardie 2002). To colonize new plants, alate morphs achieve host plant selection through a sequence of behaviors as described by Niemeyer (1990) and Powell et al. (2006). All along this sequence, the plant may be rejected at any step. If the aphid accepts the plant, settlement occurs and apterous individuals are produced. In turn, apterous individuals are involved in small—scale dispersal by walking from a plant to neighboring ones (Harrington and Taylor 1990; Boiteau 1997; Lombaert et al. 2006; Narayandas and Alyokhin 2006) in response to increasing population density or host plant quality variation (Kindlmann and Dixon 1996; Mashanova et al. 2008).

Within species, morph effect on host plant acceptance and feeding has been studied in gynoparae *Aphis fabae* (Powell and Hardie 2001), and males and apterous virginoparae of *Myzus persicae* (Hemiptera: Aphididae) (Margaritopoulos et al. 2004). Both studies revealed that phloem sap ingestion is reduced for alates on their secondary host plant (summer host for gynoparae and males) while xylem uptake increased. Higher xylem ingestion is also reported in alate compared to apterous virginoparae in *Acyrtosiphon pisum* and in *A. fabae* on *Vicia faba* (MacKay and Downer 1979; Spiller et al. 1990; Powell and Hardie 2002).

The potato crop *Solanum tuberosum* L. (Solanales: Solanaceae) is often colonized by two aphid pests, *M. persicae* and *Macrosiphum euphorbiae* Thomas (Hemiptera: Aphididae). These two aphid species present different association history with the *Solanum* genus, which comprises about 230 species distributed from the southwestern USA through Mexico, Central America, Peru, Bolivia, and extending all the way to southern South America (Hawkes 1990; Hijmans and Spooner 2001). *Macrosiphum euphorbiae* is a polyphagous species that feeds on 200 plant species belonging to 20 different families. It originated from North America, and its ability to exploit various species from the *Solanum* genus suggests a long association with *Solanum* species, one of its preferential secondary hosts (Flanders et al. 1992; Le Roux et al. 2010). The body length of *M. euphorbiae* is from 1.7 to 3.6 mm for apterae, and from 1.7 to 3.4 mm for alates (Blackman and Eastop 2000). Probably native to Asia, *M. persicae* is a widespread and polyphagous aphid that can colonize hundreds of plant species from 40 different families (Flanders et al. 1992; Blackman and Eastop 2000). Its association with *Solanum* sp. is considered more recent than that of *M. euphorbiae* (Flanders et al. 1992; Le Roux et al. 2010). The body length of *M. persicae* is from 1.2 to 2.1 mm for both apterous and alate morphs (Blackman and Eastop 2000).

Despite the economic impact of these two aphid species, a comparative study of their probing activity has not been done on a common secondary host plant, *S. tuberosum*. Owing to variations in body length between morphs and species and difference in the evolutionary history of each species, we hypothesized that probing activity would be different between alate and apterous virginoparous morphs and between *M. persicae* and *M. euphorbiae* species. Thus, the objectives of this study were to assess both their ability to remain on a potato leaflet by using a non—choice bioassay and their feeding behavior by electrical penetration graph experiments.

## Materials and Methods

### Plants and insects

Potato plants *S. tuberosum* were grown from tubers during five weeks in 90 mm plastic pots under controlled greenhouse conditions (20 ± 1°C, 60 ± 5% RH, 16:8 L:D). *Myzus persicae* and *M. euphorbiae* were reared separately on potato plants enclosed in ventilated Plexiglas® cages in two different growth chambers under 20 ± 1°C, 60 ± 5% RH, and 16:8 L:D to induce parthenogenesis. *Myzus persicae* colony was initiated from a single virginoparous female collected in 1999 in a potato field in northern France. *Macrosiphum euphorbiae* colony was established in 2003 from a single apterous parthenogenetic female from the clone MeLB05 (INRA-INSA, France, Villeurbanne).

Aphid colonies were synchronized by removing all alates two days before experiments conducted in 2010. Alate aphids in their dispersal phase were collected on the inner wall of the rearing cages, and because of their variable propensity to fly or probe they were standardized in a Plexiglas® chamber (305 mm high, 152 mm diameter) as described by Brunissen et al. (2009). Aphids were placed inside a Plexiglas® chamber in a cylindrical glass receptacle (5 mm high, 20 mm diameter). They were placed at a height of 40 mm in the center of a Petri dish (9 mm deep, 40 mm diameter) filled with water to prevent aphids leaving the receptacle without engaging in flight. A piece of yellow cardboard attached to a bamboo stem was placed in the chamber to mimic leaves. 60 to 90 min later, only alates present on the inner side of the Plexiglas® chamber or on yellow cardboard were collected and used for experiments. Apterous adult females used for experiments were 2–3 days old and collected from the rearing.

### Aphid body length

For each species and morph, 30 aphids were measured under a microscope equipped with a micrometric ocular. Aphid body length was measured in dorsal view from the center of the frons to the end of the abdomen excluding the cauda.

### No—choice bioassays

Alate and apterous aphids were individually placed with a small paintbrush on the center of the abaxial face of a potato leaflet freshly excised from a 4–5 week old whole plant and stuck by its adaxial face on a 1.5% agar poured in a Petri dish (55 mm diameter). Aphid location (i.e., on or off of the leaflet) and probing activity (i.e., mouthparts contacting the leaflet) were noted every 15 min for two hours. Mouthpart in contact with the leaflet was considered as a proxy for probing activity. For each aphid species and morph, 30 replicates were done with different individuals and statistical analysis was performed only for individuals remaining on leaflet.

**Figure 1.**
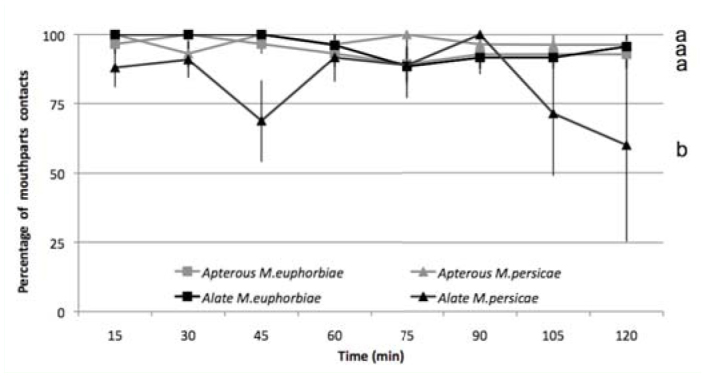
Percentage of mouthparts of apterous and alate *Macrosiphum euphorbiae* and *Myzus persicae* in contact with a potato leaflet among those remaining on it High quality figures are available online.

### EPG experiments

The DC-electrical penetration graph (EPG) technique (Tjallingii 1978) was used to investigate the feeding behavior of aphids. A gold wire (20 µm diameter, 20 mm long) was stuck with conductive water—based silver glue on the aphid dorsum. The aphid was then connected to the DC-EPG amplifier and carefully placed on the abaxial face of the third fully expanded leaf of a potted 4–5 week old potato plant. A second electrode was inserted into the soil to complete the electrical circuit. The recordings were performed during daytime for four continuous hours. Acquisition and analysis of the EPG waveforms were carried out with PROBE 3.5 software (EPG Systems, www.epgsystems.eu). The EPG-Calc 4.8 software (Giordanengo 2009) was used to calculate parameters from the recorded EPG waveforms. Seventeen parameters assigned into five classes were used to describe feeding behavior (Table 1): the general probing behaviour, the pathway phase, the salivation phase, the phloem ingestion phase, and a class related to other parameters. For each aphid species and morph, 20 replicates were done with 20 different individuals.

**Table 1.**
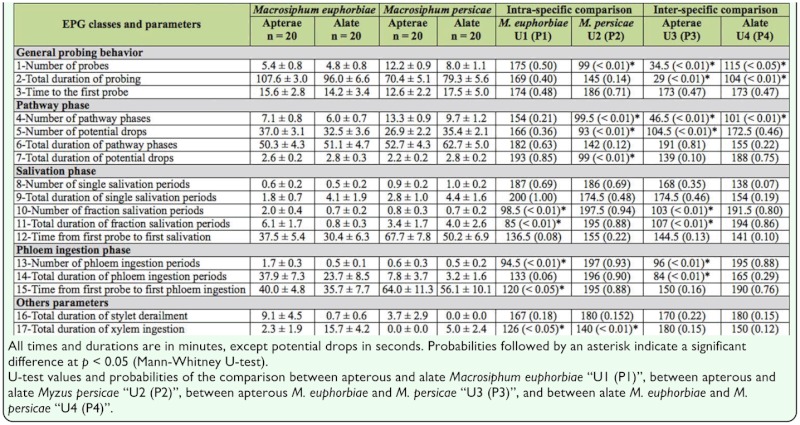
Feeding behavior (mean ± SE) of apterous and alate *Macrosiphum euphorbiae* and Myzus *persicae* characterized by 17 parameters classified in five EPG classes.

### Data analysis

Statistical analyses were performed using STATISTICA 6.0 software (StatSoft, www.statsoft.com). Differences in aphid body length were analysed using a Student's *t*-test. Non—choice data were analysed with a Pearson's Chi-squared test. Because EPG data were not normally distributed, pairwise comparisons were done between parameters of alate and apterous morphs of each species with a Mann-Whitney U-test. Significant differences were determined at *p* < 0.05.

## Results

### Aphid body size

**Intra—specific comparison.** Apterous *M. euphorbiae* mean body length (2.25 ± 0.20 mm) was higher than that of alates (2.00 ± 0.16 mm) (*t* = 5.40, df = 58, *p* < 0.01). No significant difference was found between the two morphs of *M. persicae* (apterae: 1.28 ± 0.23 mm; alates: 1.36 ± 0.16 mm; *t* = -1.57, df = 58, *p* = 0.12).

**Inter—specific comparison.** Both morphs of *M. euphorbiae* showed a higher mean body length than that of their relative *M. persicae* morphs (apterae: *t* = 17.80, df = 58, *p* < 0.01; alates: *t* = 15.38, df = 58, *p* < 0.01)

### No—choice bioassays

**Intra—specific comparison.** At the start of the bioassay, 30 individuals of each morph and species were laid on a potato leaflet. After two hours, 28 apterous and 23 alate *M. euphorbiae* remained on the leaflet (χ^2^ = 3.41, df = 1, *p* = 0.84). During the bioassay, 88 to 100% of alate and apterous *M. euphorbiae* had mouthparts contacting the leaflet, and there was no significant difference between the two morphs (χ^2^ = 2.75, df = 7, *p* < 0.907) (Figure 1). After two hours, 27 apterous and five alate *M. persicae* remained on the leaflet (χ^2^ = 76.45, df = 1, *p* < 0.91). Apterous *M. persicae* exhibited higher mouthpart contact with the leaflet than alates, especially during the last 30 min of the experiment (χ^2^ = 88.3, df = 1, *p* < 0.01). Among alate *M. persicae* that remained on the leaflet, 60 to 100% had mouthparts contacting the leaflet (Figure 1).

**Inter—specific comparison.** After two hours, both *M. euphorbiae* morphs (28 apterous and 23 alates) and apterous *M. persicae* (27 apterous and 5 alates) remained on the leaflet. A significant difference was only reported between alates of the two species (χ^2^ = 59.95, df = 1, *p* < 0.01). Mouthpart contact with the leaflet was more numerous in *M. euphorbiae* than in *M. persicae* alates (χ^2^ = 66.7, df = 1, *p* < 0.01), and no significant difference was observed between apterous morphs (χ^2^ = 0.57, df = 1, *p* < 0.999).

### EPG experiments

**Intra—specific comparison.** None of the EPG parameters from the general probing behavior and pathway phase classes differed between alate and apterous *M. euphorbiae* (Table 1). The number and the total duration of fraction salivations (i.e., salivation periods followed by phloem ingestion) were higher in apterous adults than in alates. For the apterous morph, the number of phloem ingestion (parameter 13) was higher, as well as the time from first probe to first phloem ingestion (parameter 15) compared to alates. The total duration of xylem ingestion phase was reduced for apterous *M. euphorbiae*.

Neither salivation nor ingestion phases differed between apterous and alate *M. persicae*. For this species, apterous individuals showed a higher number of probes and pathway phases (i.e., duration of stylets transit excluding xylem and phloem phases) (parameters 1 and 4) than alate ones, and number and duration of potential drops (i.e., intracellular punctures during pathway phases) (parameters 5 and 7) were reduced. Contrary to alates, apterous *M. persicae* did not exhibit xylem activity (parameter 17).

**Inter—specific comparison.** Apterous *M. persicae* probed potato leaflets more frequently than apterous *M. euphorbiae* (Table 1). Compared to *M. persicae*, both morphs of *M. euphorbiae* showed higher total duration of probing and lower number of probes and pathway phases (parameters 1, 2, and 4). Regarding parameters linked to salivation phase, the number and the total duration of fraction of salivation phases (parameters 10 and 11) were higher for apterous *M. euphorbiae* than for *M. persicae*. The number and the total duration of phloem ingestion (parameters 13 and 14) were also increased in apterous *M. euphorbiae* compared to *M. persicae*. Comparing alate aphids, the total duration of probing (parameter 2) was higher for *M. euphorbiae* than *M. persicae*, and the number of probes and pathway phases (parameters 1 and 4) were reduced for the latter species.

## Discussion

Large differences in the probing behaviors were observed between *M. persicae* and *M. euphorbiae* on *S. tuberosum*, which has been reported as a susceptible host for both species (Le Roux et al. 2007). Such variations also occurred between apterous and alate morphs of each species. Both *M. euphorbiae* morphs and only apterous *M. persicae* remained on the potato leaflet and exhibited mouthpart contact with it, suggesting a propensity for rapidly accepting their host plant. Alate *M. persicae* realized a brief gustative probe and quickly left the leaflet. Such behavior could be interpreted as a decrease in plant acceptance. Alyokhin and Sewell (2003) showed that apterous *M. euphorbiae, M. persicae*, and *Aphis nasturtii* do not leave potato plants that offer a suitable food supply. Alvarez (2007) reported that *M. persicae* did not leave *S. tuberosum* leaflets during the first two hours after landing. In our EPG study, apterous and alate *M. euphorbiae* rapidly accepted the leaflet and probed the plant (i.e., long duration of probing, phloem sap salivation and ingestion). *Solanum tuberosum* is an adequate host for *M. euphorbiae*. Thus, perceived physical and chemical cues stimulate food uptakes (Alvarez 2007).

Containing few solutes, xylem sap has high water content and can therefore be an important source of water for aphids (Gollan et al. 1992; Powell and Hardie 2002). Alates tend to reach xylem tubes to rehydrate and replenish their water balance after their dispersal flight (Powell and Hardie 2001). MacKay and Downer (1979) and Margaritopoulos et al. (2004) also showed an increased xylem uptake for alates. Although their work was done on males and gynoparae of different species (*A. fabae, A. pisum, M. persicae*), they concluded that large xylem ingestion was related to aphid morph. In *M. euphorbiae*, xylem ingestion was linked to age in both alate and apterous morphs (Pompon et al. 2010). In this study, higher xylem consumption for alate morphs of both species was also demonstrated. Surprisingly, our work revealed that alate and apterous morphs behaved differently, particularly for the general probing behavior, the pathway phase, and for the salivation and ingestion phases. In *M. persicae*, differences concerned classes related to general probing behavior and pathway phase. The important number of probes and pathway phases for apterous morphs, which correspond to aphid stylets searching for phloem cells (Tjallingii and Hogen-Esch 1993; Ramirez and Niemeyer 2000), may be interpreted as difficulties to access phloem tubes. In *M. euphorbiae*, differences concerned classes related to salivation and phloem ingestion phases. Aphid feeding times increased with body size (Douglas 2003) both for nymphal development and among aphid species (Dixon 1998). The increased phloem ingestion duration in *M. euphorbiae* apterous compared to alate was linked not only to their difference in food requirement as reported by Mittler (1973), but also to their difference in body size. The lack of difference in *M. persicae* morphs sharing a similar size, and the higher time spent in ingesting phloem by *M. euphorbiae*—which is larger than *M. persicae*—support this finding. Although *M. persicae* and *M. euphorbiae* belong to the large Macrosiphini lineage, their phylogenetic distance within this taxon is important (Von Dohlen et al. 2006). According to Jermy (1993), host plant specialization could be linked to sensory perception: generalists have evolved from specialists, losing their sensitivity to many deterrent compounds and becoming non—receptive to deterrent compounds, which normally stimulate the feeding or oviposition behavior of specialists. This could be the case with *M. euphorbiae*, which is thought to share a long association with *Solanum* plants. Despite its high polyphagous status, this aphid seems to exhibit a higher preference towards *Solanum* plants than that of *M. persicae*. Karley et al. (2002) showed that several potato cultivars are not optimal hosts for this latter species for which the broad host plant range implies a lower degree of specialization (Blackman and Eastop 2000; Dixon 1998).

Owing to differences observed on probing behavior and to similarities in plant exploitation strategies, the different length of the association between these two polyphagous aphids and the *Solanum* species could have played a major role in their preference for Solanaceae. For instance, Le Roux et al. (2008, 2010) reported that high resistance of several wild *Solanum* accessions against *M. persicae* is a phloem—based antixenosis, while it is highly variable and of various nature and location against *M. euphorbiae*. It is although possible that the closer and longer association of *M. euphorbiae* with the *Solanum* species (Flanders et al. 1992) have contributed to its feeding preference toward cultivated Solanaceae and more particularly *S. tuberosum*.

Screening a large number of clones of these two aphid species with different sizes and from different geographic areas could provide valuable findings to assess the respective influence of body size and available plant spectrum on aphid feeding behaviors.

## Abbreviations

EPG: electrical penetration graph
